# A Rare Case Report of Thoracic Ectopia Cordis: An Obstetrician's Point of View in Multidisciplinary Approach

**DOI:** 10.1155/2016/5097059

**Published:** 2016-11-09

**Authors:** Diana Ramasauskaite, Vilija Snieckuviene, Viktorija Zitkute, Ramune Vankeviciene, Dalia Lauzikiene, Grazina Drasutiene

**Affiliations:** ^1^Clinic of Obstetrics and Gynecology, Faculty of Medicine, Vilnius University, LT-03101 Vilnius, Lithuania; ^2^Faculty of Medicine, Vilnius University, LT-03101 Vilnius, Lithuania; ^3^Clinic of Children's Diseases, Faculty of Medicine, Vilnius University, LT-03101 Vilnius, Lithuania

## Abstract

Ectopia cordis is a rare congenital anomaly associated with the heart positioned outside of the thoracic cavity either partially or completely. It can be associated with other congenital abnormalities. Overall, the prognosis for infants with ectopia cordis is very poor but depends greatly on the type and severity of ectopia cordis and intracardiac and associated malformations. We present one case of a fetus with prenatally diagnosed thoracic ectopia cordis with intracardiac defects and omphalocele, all the abnormalities seen in pentalogy of Cantrell except a diaphragmatic defect. Considering poor prognosis for fetus, conservative management of prenatal care has been chosen. At the 42nd gestational week, during the active stage of labor, due to fetal distress, cesarean section was performed at a tertiary level hospital. The condition of the infant was impairing rapidly and the newborn succumbed within 24 hours. We discuss the perinatal care concerning this rare anomaly.

## 1. Introduction

Ectopia cordis is defined as an anomaly in which the fetal heart partially or completely lies outside the thoracic cavity. It is a rare congenital abnormality with an incidence of 5.5 to 7.9 per 1 million live births and includes 0.1% of congenital heart diseases [1–6]. The ectopic heart is one of the five characteristic abnormalities seen in patients presenting with the rare syndrome of pentalogy of Cantrell which comprises midline supraumbilical abdominal wall defect, deficiency of the anterior diaphragm, defect of the lower sternum, defect in diaphragmatic pericardium, and congenital heart disease [7, 8]. Clinically, ectopia cordis has been classified into four types according to the cardiac location: cervical (3% of cases), thoracic (60% of cases), abdominal (30% of cases), and thoracoabdominal (7% of cases) [6, 9, 10]. Here we report one thoracic ectopia cordis case with anterior abdominal wall defect, supraumbilical omphalocele, heart ectopia, and congenital intracardiac defects.

## 2. Case Report

A 32-year-old woman, gravida 5 para 2, had no regular prenatal care before 24 weeks of gestation. An anterior thoracic defect with an extrathoracic two-chamber heart was recognized at 24 gestational weeks of uneventful pregnancy during the first ultrasonographic evaluation. There was no family history of congenital anomalies, genetic abnormalities, or history related to ectopia cordis. During pregnancy, the mother was smoking 15 cigarettes per day. An unfavorable prognosis to the fetus was predicted and conservative management of prenatal care had been chosen.

**Figure 1:**
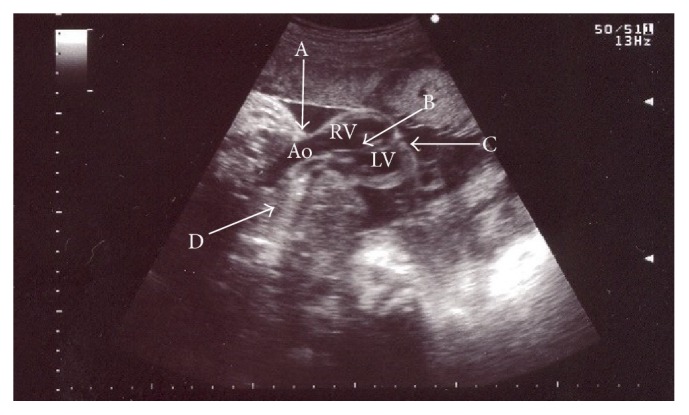
Ultrasound of fetus heart at 42nd week of gestation, thoracic cleft, and major blood vessels transposition. Ao: aorta, RV: right ventricle, and LV: left ventricle. A: wide anterior thoracic defect, B: ventricular septum, C: ectopic heart, and D: chest.

**Figure 2:**
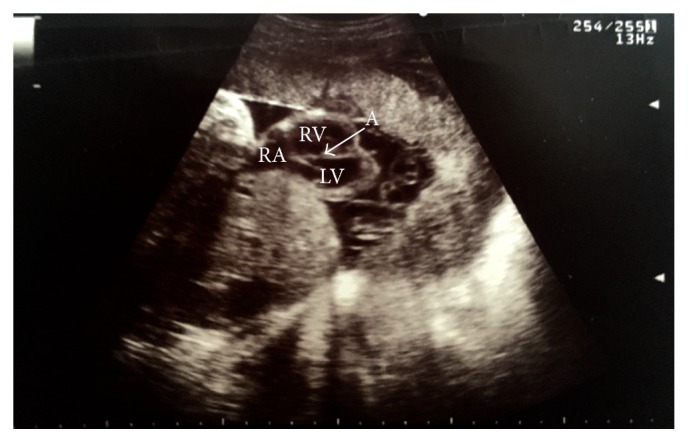
Ultrasound of fetus heart at 42nd week of gestation with ventricular septal defect. RA: right atrium, RV: right ventricle, and LV: left ventricle. A: ventricular septal defect.

**Figure 3:**
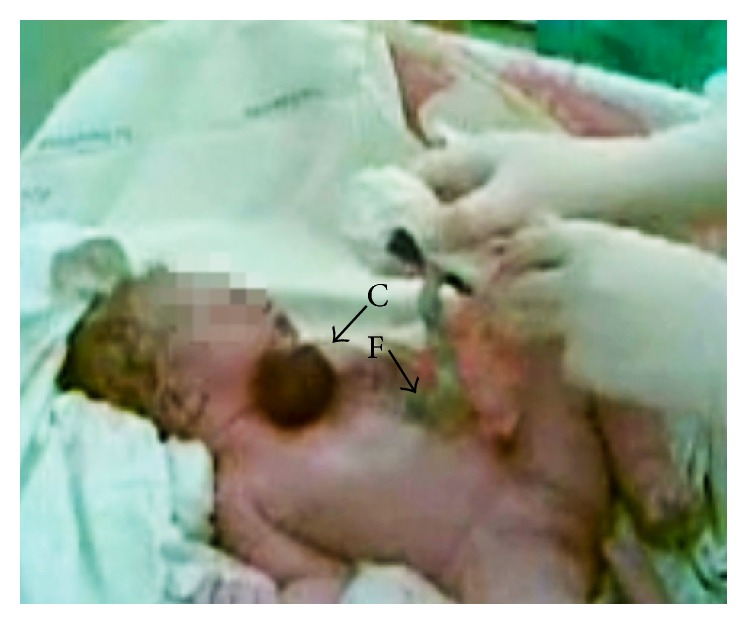
The newborn 5 minutes after birth. C: the ectopic heart positioned outside the thoracic cavity; F: supraumbilical omphalocele.

The woman was admitted to the tertiary level obstetrics and gynecology clinic during active stage of labor, at gestational age of 42 weeks. Diagnosis of congenital heart anomaly was heart ectopia, and septal ventricular defect was confirmed by ultrasound. Multiple anomalies were observed: wide anterior thoracic defect with extrathoracic four-chamber heart, rounded apex of the heart, high ventricular septal defect, the major blood vessels transposition, narrow pulmonary artery, and pericardium covering only ventricles (Figures 1 and 2). Despite unfavorable prognosis to the fetus, mother had chosen intrapartum fetal heart monitoring. Due to fetal distress at a cervical dilation of 6 cm, cesarean section was performed. The newborn was a female of 3300 g weight and 44 cm height who scored 8 (1 min) and 8 (5 min) on Apgar scale (Figure 3, Supporting Information Video 1 in Supplementary Material available online at http://dx.doi.org/10.1155/2016/5097059). At birth, the infant had hypotonia, weak cry, and generalized facial cyanosis. The physical examination revealed split sternum with complete thoracic ectopia cordis, the defect followed by anterior abdominal wall defect, supraumbilical omphalocele. Ectopic heart with partial absence of the pericardium was beating outside the thoracic cavity, at a rate of 130/min with remittent bradycardia. After birth, the infant's heart was covered with warm saline-soaked sterile dressing. The newborn girl was transferred to the specialized cardiac surgery centre, children's intensive care unit. She died within 24 hours. The parents declined postmortem newborn's autopsy.

## 3. Discussion

Ectopia cordis (EC) is a rare and impressive congenital malformation, which was observed thousands of years ago. The term “ectopia cordis” has been used to describe all anomalies in which the heart was not located within the thorax [1]. The etiology of this pathology has not been fully explained. Embryologically, it is caused by failure of lateral mesoderm in the third week of intrauterine life and failure of midline fusion of the developing chest wall caused by compression of the thorax resulting from rupture of the chorion or yolk sac at around 21 days of gestation [10]. However, the relationship between abnormal karyotypes such as XXY, trisomy 18, and trisomy 21 was observed [6, 10].

Ultrasound made as early as within the first trimester or by the start of the second trimester allows enough time to define the associated abnormalities in nearly 90% of the reported cases [9–11]. Ectopia cordis and large omphalocele are detected with 2D ultrasonography, which is commonly sufficient in diagnosis [12]. However, sometimes this may be difficult particularly in minor forms of ectopia cordis. Then, it is better to use 3D scanning to visualize fetal bones due to greater contrast difference compared with contiguous organs [1, 12, 13]. Fetal cardiac MRI has the potential to offer an alternative imaging option in patients to whom echocardiography is limited by maternal or fetal factors (e.g., maternal obesity, adverse fetal position, and placental calcifications). Also it is highly recommended to perform the chromosomal analysis due to the association with aneuploidy, especially trisomy 18 [9, 12].

Overall the prognosis of ectopia is very poor, but depends greatly on the type and severity of EC, intracardiac and associated malformations, gestational week, birth weight, mode of delivery, or even by the available medical resources [14–17]. Cervical ectopia cordis is completely incompatible with life [7]. Thoracic type is related to poor outcome. The majority of long-term survivors with thoracic type had no associated cardiac defects. Engum has reported only one surviving newborn from 91 cases with true thoracic ectopia cordis and intracardiac defects, similar to our case [17]. The other types of ectopia cordis have better prognosis with multiple reports of successful elective repairs into infancy and early childhood [10]. Untreated, this kind of anomaly is fatal and most infants are stillborn or die within the first few hours or days of life from infection, cardiac failure, or hypoxemia [14, 17].

Perinatal care when parents chose sustaining the pregnancy or termination is impossible because gestational age greater than 22 weeks has been rarely discussed in the literature. To offer the best care and therapy, a multidisciplinary medical team consisting of a perinatologist, a neonatologist, a radiologist, a pediatric surgeon, a cardiologist, a pediatric cardiac surgeon, a plastic surgeon, and palliative nurses should counsel parents preferably prenatally. Therefore, it is necessary to diagnose the severity of pathology and estimate the prognosis to fetus as precisely as possible to inform the family with frankness. Provided with accurate medical information about the delivery mode and possible infant care, the parents should decide autonomously. Decisions regarding fetal monitoring and mode of delivery are difficult. Parents choosing vaginal delivery should be informed about the possibility of fetus demise during labor due to prolonged cardiac compression, damage of herniated viscera, or rupture of an atrial diverticula or omphalocele sac [17]. They also should know, that performing cesarean section does not often change the outcome [2, 10]. In our case, the conservative approach was offered, but during the labor it was changed to active labor management plan because of patient request. Due to fetal condition, cesarean section had been performed, but it did not have an impact on the previously determined poor outcome.

In conclusion, early prenatal detection and precise diagnosis of ectopia cordis are essential for multidisciplinary team to provide optimal parental counselling for fetus/infant prognosis, aiding in developing a delivery plan and, when possible, postnatal management strategy.

## Supplementary Material

The newborn female at birth, presented with wide thoracic defect, four chambers heart with partialy absent pericardium and round-shaped apex was beating outside the thoracic cavity. This defect was followed by anterior abdominal wall defect, supraumbilical omphalocele.

## References

[1] Chelli D., Dimassi K., Jallouli-Bouzguenda S. (2008). Prenatal diagnosis of ectopia cordis: case report. *Tunisie Medicale*.

[2] Kabbani M. S., Rasheed K., Mallick M. S., Abu-Hassan H., Al-Yousef S. (2002). Thoraco-abdominal ectopia cordis: case report. *Annals of Saudi Medicine*.

[3] Zhang X., Xing Q., Sun J., Hou X., Kuang M., Zhang G. (2014). Surgical treatment and outcomes of pentalogy of Cantrell in eight patients. *Journal of Pediatric Surgery*.

[4] Sadłecki P., Krekora M., Krasomski G. (2011). Prenatally evolving ectopia cordis with successful surgical treatment. *Fetal Diagnosis and Therapy*.

[5] Apte A. V. (2008). Thoraco-abdominal ectopia cordis: a rare entity. Case report and review of literature. *People's Journal of Scientific Research*.

[6] Çelik Y., Hallıoğlu O., Basut N., Demetgül H., Kibar A. E. (2015). A rare case of cardiac anomaly: prenatally diagnosed ectopia cordis. *Turk Pediatri Arsivi*.

[7] Malik R., Zilberman M. V., Tang L., Miller S., Pandian N. G. (2015). Ectopia cordis with a double outlet right ventricle, large ventricular septal defect, malposed great arteries and left ventricular hypoplasia. *Echocardiography*.

[8] Harring G., Weil J., Thiel C., Schmelzle R., Mueller G. C. (2015). Management of Pentalogy of Cantrell with complete ectopia cordis and Double Outlet Right Ventricle. *Congenital Anomalies*.

[9] Sepulveda W., Wong A. E., Simonetti L., Gomez E., Dezerega V., Gutierrez J. (2013). Ectopia cordis in a first-trimester sonographic screening program for aneuploidy. *Journal of Ultrasound in Medicine*.

[10] Gabriel A., Donnelly J., Kuc A. (2014). Ectopia cordis: a rare congenital anomaly. *Clinical Anatomy*.

[11] Shad J., Budhwani K., Biswas R. (2012). Thoracic ectopia cordis. *BMJ Case Reports*.

[12] Ergenoğlu M. A., Yeniel A. Ö., Peker N. (2012). Prenatal diagnosis of Cantrell pentalogy in first trimester screening: case report and review of literature. *Journal of the Turkish German Gynecology Association*.

[13] Chang Y., Yang M.-J., Wang P.-H., Chen C.-Y. (2015). Three-dimensional HDlive image of ectopia cordis in a twin fetus at 9 gestational weeks. *Taiwanese Journal of Obstetrics and Gynecology*.

[14] Taksande A. M., Vilhekar K. Y. (2013). A case report of ectopia cordis and omphalocele. *Indian Journal of Human Genetics*.

[15] Chishugi J. B., Franke T. J. (2014). Thoraco-abdominal ectopia cordis in southwest cameroon. *Pan African Medical Journal*.

[16] Morello M., Quaini E., Nenov G., Pomé G. (1994). Extrathoracic ectopia cordis: case report. *Journal of Cardiovascular Surgery*.

[17] Engum S. A. (2008). Embryology, sternal clefts, ectopia cordis, and Cantrell's pentalogy. *Seminars in Pediatric Surgery*.

