# Supplementary material for: A Rare Case Report of Thoracic Ectopia Cordis: An Obstetrician's Point of View in Multidisciplinary Approach
Source: Case Rep Pediatr. 2016 Nov 9;2016:5097059. doi: 10.1155/2016/5097059 (PMC5120199; doi:10.1155/2016/5097059)

Supporting information Video 1. Thoracic ectopia cordis, the heart beating outside the infant’s thoracic cavity.


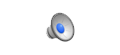

Supplement: Supplementary file 2 [file 5097059.f2.doc]
